# Biomanufacturing and Scale‐Up: Pathways to Biochemicals, Biofuels, and Biomaterials

**DOI:** 10.1111/1751-7915.70425

**Published:** 2026-08-07

**Authors:** Carolina A. Barcelos, Pavel Dvořák, Charles J. Foster, Juan Nogales, Juan L. Ramos, Shamlan M. S. Reshamwala, Davinia Salvachúa

**Affiliations:** ^1^ Advanced Biofuels and Bioproducts Process Development Unit Lawrence Berkeley National Laboratory Emeryville California USA; ^2^ Department of Experimental Biology, Faculty of Science Masaryk University Brno Czech Republic; ^3^ Biotechnology Bioprocess Improvement DSM‐Firmenich Columbia Maryland USA; ^4^ Department of Systems Biology Centro Nacional de Biotecnología (CSIC) Madrid Spain; ^5^ Estación Experimental del Zaidín (CSIC) Granada Spain; ^6^ Institute of Chemical Technology Mumbai India; ^7^ Renewable Resources and Enabling Sciences Center National Laboratory of the Rockies Golden Colorado USA

## 1 Special Issue: Advances in Biomanufacturing and Scale‐Up for Biochemicals, Biofuels, and Biomaterials

Advancing the bioeconomy requires the development of large‐scale microbial bioprocesses capable of converting *waste* carbon streams into biofuels, biochemicals, and biomaterials at industrially relevant scales. While biomanufacturing has been successfully demonstrated at the laboratory scale for a wide range of chemicals, only a few have reached industrial‐scale production. This is partly due to the inherent complexity of microbial systems, which rely on living cells with intricate metabolic pathways that are highly sensitive to environmental changes, making large‐scale production difficult to optimize and predict. As a result, scaling‐up bioprocesses remains a high‐stakes challenge that requires deeper exploration. This involves integrating feedstock and microbial selection, upstream and downstream processes, and computational modelling, among other research efforts. Bulk and specialty chemicals derived from biological processes also face competition from fossil‐based production routes, which have been refined through decades of technological advancements. While biologically derived molecules may offer more environmentally friendly production pathways than traditional chemical manufacturing, their widespread adoption depends on achieving cost parity—or superiority—relative to fossil‐based methods. This emphasizes the importance of holistic research, including techno‐economic analyses and life cycle assessments, to ensure both economic viability and environmental sustainability.

This editorial and special issue explores state‐of‐the‐art strategies for converting *waste* carbon sources into valuable products. It discusses how enzymes, single microbes (e.g., extremophiles), and microbiomes (e.g., through division of labor) can be integrated with upstream and downstream process innovations—such as consolidated bioprocessing and in situ product recovery—to improve the efficiency and scalability of biomanufacturing. The editorial further highlights the role of computational modelling in understanding, predicting, and controlling bioprocess performance across scales, and concludes by emphasizing the importance of techno‐economic modelling to identify technologies that can move to market.

## 2 Biocatalysts and Bioproducts

Advancing the bioeconomy will depend on the ability to convert *waste* carbon streams—including lignocellulose, organic waste (e.g., municipal solid waste), synthetic waste (e.g., plastics), and C1 and C2 feedstocks—into biochemicals, biofuels, and biomaterials. This special issue includes a series of articles on promising biocatalysts, including microbes and enzymes, toward the production of valuable products from various carbon sources and their use in biotechnological applications.

An industrially value‐added compound is the organic acid L‐(+)‐tartaric acid, which is currently produced using a combination of chemical and enzymatic synthesis from petroleum‐derived C4 feedstocks. A fully biological route for production of tartaric semialdehyde, a precursor of L‐(+)‐tartaric acid, from glucose is described by Li et al. (2025) in this special issue. By expressing a transketolase in the metabolically versatile *Gluconobacter deoxydans*, a “Push‐Pull” strategy was implemented to increase flux toward 5‐keto‐D‐gluconic acid, a key intermediate. In 5 L bioreactor studies, 48.88 ± 2.16 g/L tartaric semialdehyde was produced by the plasmid‐free genome integrated strain from 100 g/L glucose, demonstrating feasibility of L‐(+)‐tartaric acid production using renewable resources.

Polyketides, characterized by their structural diversity, have also broad utility across multiple industries. Polyketide production by (bacterial) actinomycetes typically occurs during the stationary phase, where decreased acyl‐CoA availability limits titres required for economically viable production. In this special issue, Yang et al. (2024) report engineering *Streptomyces avermitilis* to increase production of avermectin B1a, a macrocyclic polyketide with strong insecticidal activity. Expression of a newly designed polyketide synthase increased synthesis of the starter unit, 2‐methylbutyryl‐CoA, and CRISPRi‐mediated inhibition of three key nodes in essential pathways increased levels of the extender units malonyl‐CoA and methylmalonyl‐CoA. Combined with overexpression of fatty acid β‐oxidation pathway genes from the high‐yield parental *S. avermitilis* A229 strain, these novel strategies led to a B1a titre of 8.8 g/L, representing a 37.8% increase. Enhancing precursor supply represents a potential strategy for increasing the production of other polyketides.

Plastic pollution is widely recognized as an intractable problem requiring urgent, viable solutions. In this special issue, Op de Hipt et al. (2025) used a combination of adaptive laboratory evolution and reverse engineering to expand the substrate range of an aromatics‐overproducing *Pseudomonas taiwanensis* strain to adipate and 1,4‐butanediol, monomers that make up the biodegradable aliphatic aromatic co‐polyester poly(butylene adipate‐co‐terephthalate). A gene encoding a tyrosine ammonia‐lyase, which converts tyrosine to 4‐coumarate, a potential building block for new synthetic polymers, was expressed to produce 4‐coumarate from adipate and 1,4‐butanediol. This work contributes to the advancement of a circular plastics economy.

Enzymatic strategies also provide efficient and stereoselective routes for the synthesis of optically pure compounds. N‐Acyl‐D‐amino acid deacylases (also known as D‐acylases) of different enantiospecificities coupled to an N‐succinyl‐amino acid racemase (NSAR) are used to synthesize different optically pure D‐ and L‐amino acids. In this special issue, Martínez‐Rodríguez and Gavira (2025) report characterization of two new recombinant D‐acylases from *Bordetella petrii* and *Klebsiella pneumoniae* . When coupled with an NSAR from *Geobacillus stearothermophilus* , biosynthesis of D‐methionine or D‐aminobutyric acid was demonstrated. Structural analysis of the D‐acylase of *K. pneumoniae* provides only the second experimentally determined 3‐D structure of a member of this enzyme family.

Biodesulfurization, as a biocatalytic alternative to hydrodesulfurization for removing sulfur compounds from crude oil, exemplifies the expanding role of microbes and enzymes in industrial processes. To overcome the challenges associated with biodesulfurization, Glekas et al. (2025) describe genome engineering strategies to optimize a genome‐reduced *Pseudomonas putida* EM384 strain to remove sulfur atoms from the model heterocyclic sulfur‐containing component of raw petroleum, dibenzothiophene. The *dsz* operon of the *Rhodococcus qingshengii* IGTS8, which is naturally capable of degrading dibenzothiophene, was reordered and engineered to increase its transcription and translation efficiency, and the resulting optimized operon was integrated into the *P. putida* EM384 genome. These genetic modifications led to high desulfurization efficiency in model biphasic biodesulfurization systems.

## 3 Consolidated Bioprocessing and In Situ Product Recovery

Lignocellulosic biomass, derived primarily from agricultural residues, represents an abundant feedstock for bioproducts generation. The efficient conversion of lignocellulosic biomass into fermentable sugars still remains a central challenge in biorefinery operations, primarily due to its structural complexity. Current commercial processes face multiple technical and economic barriers that significantly impact operational viability, including complex feedstock logistics, high energy demands and enzyme costs, and considerable sugar losses during separation steps. Additional challenges arise from inefficient cellulose decrystallization, incomplete recovery of pretreatment chemicals, and the necessity for extensive wastewater treatment. These factors collectively contribute to elevated operating costs that hinder widespread commercial adoption. Success in industrial‐scale operations also requires the development of advanced microbial strains capable of efficient carbon co‐utilization while maintaining robust performance and high product yields in the presence of pretreatment‐generated inhibitory compounds.

Recent advances have demonstrated promising solutions to some of these challenges through the development of ‘one‐pot’ processes, which integrate pretreatment, saccharification, and fermentation in a single vessel. This approach is not limited to lignocellulosic biomass and can also be extended to other polymeric feedstocks, such as plastics and organic waste. By eliminating intermediate separation steps, these consolidated processes substantially reduce both capital and operating costs. The separation‐free approach can maintain high solid loading throughout the process while eliminating intermediate (e.g., sugar) losses and wastewater production typically associated with conventional separation and washing steps. Green solvents have played a crucial role in enabling efficient ‘one‐pot’ conversion of lignocellulosic feedstocks to fuels and chemicals. Remarkable results using choline‐based aprotic ionic liquids for ethanol production have been reported, demonstrating over 90% biomass conversion while maintaining compatibility with both enzymatic and microbial processes. Translating this process to larger scale, Barcelos et al. (2021) conducted a pioneering pilot‐scale demonstration that integrated ionic liquid pretreatment and fermentation. Their process achieved 83% combined pretreatment and saccharification conversion, along with > 90% fermentation efficiency for C5 and C6 sugars to ethanol by *Saccharomyces cerevisiae* . They further demonstrated 80% ethanol recovery from the whole slurry hydrolysate, achieving fermentation efficiency comparable to industrial‐scale first‐generation feedstock processes without requiring solid separation or nutrient addition, which represents a significant advancement in process integration and efficiency.

Advances in microbial platforms further expand the toolkit for consolidated bioprocessing. Filamentous fungi are emerging as powerful microbial hosts for this purpose, as they naturally couple lignocellulose depolymerization with the secretion of robust enzyme cocktails within a single organism. Among these, *Myceliophthora thermophila*, a thermophilic fungus widely established as an industrial enzyme production platform, stands out as a particularly promising chassis for integrated biorefineries, owing to its exceptional secretion capacity and thermostable carbohydrate‐active enzymes involved in lignocellulose breakdown. In this special issue, Lai et al. (2025) dissect the regulatory mechanisms controlling cellulase and xylanase expression, revealing how a single transcription factor coordinates enzyme production, cellular growth, and protein quality control. These findings provide key mechanistic insights for engineering fungal strains with improved biomass deconstruction efficiency. Continued progress in understanding and engineering fungal regulatory networks thus holds considerable promise for reducing enzyme costs and advancing the commercial viability of lignocellulosic biorefineries.

In many bioprocesses, product inhibition can also present a significant challenge, where the accumulation of toxic end‐products in the fermentation broth can severely inhibit microbial activity and limit further production. For volatile products such as ethanol, medium‐chain alcohols, esters, and monoterpenes, the integration of in situ product removal with fermentation represents an effective complementary strategy for enhancing process performance. Continuous product removal through evaporation can offer several advantages over conventional liquid–liquid extraction methods. This approach generates a high‐purity product stream while eliminating soluble contaminants present in the fermentation broth. Recent innovations in separation technologies have also demonstrated remarkable improvements in process efficiency. For example, Sun et al. (2020) developed an advanced gas stripping‐solvent scrubbing method for limonene production and recovery that enabled a five‐fold increase in productivity compared to conventional liquid–liquid extraction and a 70% reduction in the minimum selling price. Similarly, Barcelos et al. (2026) demonstrated efficient recovery of volatile products from fermentor off‐gas via condensation in a chilled solvent, achieving 84% capture efficiency and a 94% increase in product titre compared to liquid–liquid extraction.

As an additional contribution to this special issue, Loprete et al. (2025) present another product recovery strategy. Specifically, the authors developed a light‐driven cyanobacterial platform for indigo production using an engineered *Synechocystis* sp. PCC6803 strain expressing a flavin‐containing monooxygenase (mFMO), enabling photosynthesis‐fueled, light‐dependent indigo biotransformation from indole. The system achieved a high‐yield conversion of indole into indigo (up to 86%), demonstrating the potential of photoautotrophic host organisms for redox‐intensive bioprocesses. To address product inhibition and facilitate downstream processing, the authors introduced a solid‐phase recovery strategy based on polyamide‐net adsorption, which selectively captures indigo directly from the culture medium. This approach enables efficient separation of the dye from the aqueous broth, minimizes solvent consumption, and reduces environmental impact compared with conventional solvent‐intensive extraction routes. The integration of in situ product recovery via adsorption enhances both process performance and product‐stream purity, while aligning with green‐chemistry principles.

Continued advancement in key areas, including solvent recovery, separation technologies, and microbial strain engineering, will be critical for optimizing the process economics and environmental performance of future integrated and consolidated bioprocesses.

## 4 Unlocking C1 and C2 Feedstocks with Microbial Platforms

C1 and C2 substrates, used either as primary carbon inputs or as carbon and electron supplements, offer a significant but underexploited opportunity for waste carbon recovery in industrial biotechnology. A vast CO_2_ pipeline network is being planned (Doucette and Vedala 2023; Sanchez et al. 2018), and if constructed will ensure no shortage of cheap, concentrated feedstock for the production of reduced C1 and C2 compounds. Although C6 sugar feedstocks remain widely used in industry as primary carbon sources, increasing efforts are being directed toward processes incorporating C1 and C2 compounds, with significant progress in developing industrially viable routes based on ethanol and acetate.

A key advantage of either ethanol or acetate as a feedstock is in their entry‐point into metabolism; each is 2–3 enzymatic steps from acetyl‐CoA, a key precursor for innumerable bioproducts (Sun et al. 2024), with complete carbon conversion (as compared to glycolysis: 11 enzymatic steps, 2/3 carbon efficiency). The challenges with either are (broadly) stress response, substrate tolerance, and slow uptake rate. Significant efforts, therefore, have been focused toward both metabolic engineering and process development to overcome those hurdles in a number of production hosts with much success, and that effort will need to continue into the future.

Despite the associated challenges related to metabolic engineering, liquid volatility, toxicity, and/or gas solubility, progress toward developing hosts and processes using C1 compounds (methanol, methane, formic acid, CO, and CO_2_) has achieved a degree of success. Notably, a significant improvement in biomass yield has been demonstrated using glucose/formic acid co‐substrates in an aerobic yeast process at pilot scale (van Winden et al. 2022), and companies have installed anaerobic production capacity for manufacturing ethanol, acetic acid, and polyhydroxyalkanoates from waste gas streams. At the same time, academic science continues to advance our fundamental understanding of C1‐metabolizing organisms. In this special issue, Ingelman et al. (2025) advance our understanding of genotype–phenotype relationships in gas fermenting acetogen *Clostridium autoethanogenum*. By reverse‐engineering adaptive laboratory evolution isolates, they assign causality to faster auxotrophic growth and improved fermentation process robustness of adaptive laboratory evolution strains when compared to parent strains and generate insights into *C. autoethanogenum*'s regulatory network.

Outside of metabolism and/or fermentation‐centric research, process system engineers can play a pivotal role in bringing net‐zero C1 and C2 fermentation systems to scale. Coupling heterotrophic fermentation systems with CO_2_‐upgrading and green hydrogen‐producing process units to create closed‐loop aerobic systems holds potential not only for improved sustainability but economic viability.

## 5 Enhancing Bioprocess Robustness with Extremophilic Microorganisms

The effectiveness of bioprocesses needs improvement at all stages, including upstream processing, fermentation, and downstream processes. As discussed, one promising strategy is the use of ‘one‐pot’ processes. Additionally, the use of newly isolated or engineered microbial strains with enhanced robustness, which can be integrated with upstream and downstream operations, also shows great potential. Currently, industrial microbial conversions often take place under ambient conditions in terms of temperature, pH, pressure, or osmolarity. However, both upstream and downstream processes frequently require elevated temperatures, high salt concentrations, extreme pH, or pressure to break down substrates and separate products from the fermentation broth. This is especially true for recalcitrant feedstocks such as lignocellulose, certain municipal solid wastes, and synthetic plastics, which must be pretreated and depolymerized. These processes also deal with diverse hot, acidic, basic, and/or toxic waste streams. Such incompatibilities increase bioprocess costs due to the need to adjust process parameters between stages. Additionally, fermentation processes conducted under ambient conditions are susceptible to contamination and require sterilization of media, feedstock, and bioreactor infrastructure.

The optimal solution is to search for organisms from nature that are compatible with the entire process. Although challenging, promising candidates include extremophilic bacteria, archaea, and fungi. A notable example is halophilic bacterial polyhydroxyalkanoate (PHA) producers from the genus *Halomonas*. These demonstrate that combining metabolic engineering of suitable extremophilic strains with process optimization can result in high content, yield, and titre of target chemicals that meet market demands. The use of *Halomonas* strains capable of customized PHA production under non‐sterile or semi‐sterile conditions in saline environments has enabled several Chinese companies to offer bioplastics at reduced prices on the global market (Tan et al. 2021).

Thermophilic microorganisms are also seeing renewed interest in biotechnology. Thermophilic bacteria and fungi, such as *Clostridium thermocellum* , *Parageobacillus thermoglucosidasius*, and *Myceliophthora thermophila*, are no longer just donors of thermophilic enzymes but are considered promising whole‐cell catalysts for consolidated bioprocessing or simultaneous depolymerization and fermentation of crystalline lignocellulosic residues and synthetic plastics (‘one‐pot’ processes). Notably, some strains of *P. thermoglucosidasius* can also convert CO to H_2_ under aerobic conditions, which is an attractive feature for potential high‐purity hydrogen production from syngas that would not need to be cleaned of oxygen (Paredes‐Barrada et al. 2024). Bioprocesses operating at high temperatures above 45°C–50°C offer, in general, advantages such as better compatibility with “hot” waste streams and upstream processes, increased substrate solubility (excluding gases), accelerated reaction rates, and lower cooling costs compared to processes based on mesophilic hosts generating metabolic heat. There is also potential for reduced heating costs when recovering volatile products such as bioethanol.

Recent advances in thermophile research include the bacterium *Caldimonas thermodepolymerans*, a robust PHA producer capable of converting diverse lignocellulose‐derived sugars including xylose into high polyhydroxybutyrate content at 50°C, tolerating common lignocellulosic inhibitors. Grybchuk‐Ieremenko et al. (2025) report in this special issue the first dedicated genetic engineering toolbox for this Gram‐negative thermophile, including optimized electroporation, triparental mating, homologous recombination, and transposon‐based systems. They generated an engineered chassis strain with improved transformation efficiency, enabling targeted metabolic engineering, and a *phaC* deletion mutant. These tools lay the groundwork for developing *C. thermodepolymerans* into a high‐performance thermophilic PHA production host suitable for one‐pot, high‐temperature processes with reduced contamination risk and improved integration into lignocellulosic biorefineries. The ability of *C. thermodepolymerans* to degrade PHA through its potent extracellular depolymerase holds promise for closing the cycle, enabling the use of a single organism for both PHA production and biological recycling.

Another promising extremotolerant chassis is the polyextremotolerant fungal genus *Aureobasidium*, reviewed in this special issue by Xiao et al. (2024). Species such as *A. pullulans* thrive under high salinity, broad pH, and osmotic ranges, grow on low‐cost agricultural and industrial side streams, and produce a broad product portfolio including the polysaccharide pullulan (with already established large‐scale industrial production), pigments (melanin), biosurfactants (polyol lipids), or biopolymers (polymalate, β‐glucan). Their robustness offers potential for reduced sterilization, use of seawater, or valorization of saline waste streams, while their enzymes (extremozymes) have applications in food, feed, and biofuels. The review highlights advances in genome sequencing, strain characterization, initial genetic tool development, and process development paving the way for systematic strain improvement and scale‐up strategies. Such traits make *Aureobasidium* spp. valuable candidates for sustainable, cost‐effective bioprocesses in circular bioeconomy contexts.

Some other microorganisms are not extremophiles in the strict sense, but they attract attention because of their resistance to oxidative stress resulting from the processing of waste streams containing toxic chemicals. A prominent example is some members of the genus *Pseudomonas*, for example, *P. putida* KT2440. Recent studies showing the (co)processing and valorization of substrates from chemically or enzymatically degraded mixed plastic wastes, municipal wastes, or lignocellulosic residues are remarkable demonstrations of the metabolic capacity and robustness of this bacterium (Silva et al. 2025).

Recent progress in the physiological and biochemical characterization of new isolates, along with advancements in genetic and metabolic engineering tools for extremophiles over the past decade, has enabled further fine‐tuning of their biocatalytic properties and expanded their utility (Obruča et al. 2022). Advances in genome sequencing and systems biology are also enabling faster assembly of high‐quality genome‐scale metabolic models of extremophiles that help reveal metabolic bottlenecks and define minimum requirements for growth, thereby further reducing bioprocessing costs.

## 6 Advancing Industrial Biotechnology with ‘Distributed Biocatalysis’

Significant milestones have been achieved in industrial biotechnology driven by advances in systems and synthetic biology including the production of drugs, food additives, or advanced biofuels, among others. Nevertheless, meeting the societal, economic, and environmental demands of today's society underscores the need for a strategic rethinking of biotechnology approaches. The introduction of artificial intelligence (AI) and machine learning (ML)‐driven bioprocess design, as described above, along with emerging automation within iterative optimization cycles such as the Design‐Build‐Test‐Learn (DBTL) pipeline (Gurdo et al. 2023), offers a promising complement to these efforts.

In this context, the study by Duman‐Özdamar et al. (2025) demonstrates the potential of the DBTL cycle to optimize lipid production in *Yarrowia lipolytica*, providing a sustainable alternative to palm oil. During the Design phase, genome‐scale metabolic modelling was used to identify key genetic targets and bottlenecks in lipid biosynthesis. In the Build phase, genes identified in the design stage, including ACL, ACC, TS, and DGA1, were overexpressed, while the citrate exporter (CEX1) and the β‐oxidation pathway (MFE1) were disrupted. In the Test phase, amino acid supplementation was optimized and the performance of recombinant strains was evaluated. Finally, in the Learn phase, regression and interaction modelling were applied to identify the most effective engineering strategies. As a result of this DBTL cycle, the combined overexpression of TS and DGA1 in the Δmfe Δcex background resulted in a 200% increase in lipid content (56% w/w) and a 230% increase in glycerol yield. This integrated DBTL approach illustrates how iterative modelling and experimental validation can transform *Y. lipolytica* into an efficient microbial cell factory, advancing both our understanding of lipid metabolism and the development of sustainable bioprocesses.

Despite these successful approaches, as the complexity of both initial feedstocks and target products increases, it becomes evident that final titers and the economic feasibility of the given bioprocess are increasingly compromised. This is largely due to the growing metabolic burden resulting from extensive pathway engineering, limitations in cofactor availability, and the toxicity or solubility issues of intermediates and/or final products. In this context, the introduction of the division of labor (DoL) or ‘distributed biocatalysis’ concept, which promotes distributed catalysis within microbial communities, is emerging as a new paradigm in complex biotechnological bioprocesses. Pioneering efforts have successfully addressed challenges in the biosynthesis of plant secondary metabolites, the upcycling of recalcitrant polymers, and even complex biocomputing, helping to establish the foundations of the field. Additionally, the advantages of distributed catalysis over monocultures have been elegantly demonstrated in the production of PHAs from polyethylene terephthalate (PET)‐derived monomers. Despite these promising achievements and the substantial efforts to engineer functional microbiomes using both top‐down and bottom‐up approaches, current microbiome engineering still relies heavily on trial and error and/or combinatorial approaches, highlighting the need for more rational and system‐level pipelines. Computational‐driven analysis is thus emerging as a powerful tool, not only for analysing but also for the rational design of synthetic microbial communities. The development of more accurate models and the advent of AI‐driven methods (Choudhary and Mahadevan 2024) are expected to pave the way for efficient design of complex microbiomes in the near future.

Optimal microbiome engineering is not the only aspect that requires optimization to advance the field. As more efficient designs emerge, it becomes increasingly evident that the habitat and physical container for these biocatalysts—such as microbiome‐dedicated bioreactors—also require further attention. A complex microbiome demands not only a shared culture medium but also a consensus on environmental conditions to maximize functional performance (Chen et al. 2024). While traditional bioreactors have initially been used to optimize microbial consortia‐based bioprocesses, there is now a need to envision and develop a new generation of bioreactors specifically designed to drive cost‐effective microbiome‐fueled bioprocesses.

## 7 Toward Digital Twins in Bioprocess Engineering

For all of the bioprocesses detailed in the sections above, bioprocess modelling plays an integral role in bioprocess development and scale‐up, from the initial concept to the continuous improvement of commercial processes. Constructing mathematical descriptions of biological processes not only allows for the prediction of performance, but the elucidation of (oftentimes nonintuitive) mechanistic drivers that influence performance. In the past, mechanistic and empirical approaches had divergent paths, but each has yielded important insights and identified strategies for process improvement.

Mechanistic approaches have increased in complexity compared to traditional black‐box approaches, both at the intracellular level and the bioreactor scale. These advancements have resulted in computationally expensive methods that provide scientists with deeper and clearer insights into the systems they study. While the level of detail in these descriptions is often extraordinary, the associated computational expense renders many of these frameworks impractical for digital twin applications, as simulations take too long to run. In parallel, ML and AI have also matured and been applied to biological systems leading to countless insights while bypassing the need for mechanistic understanding. These empirical models are useful for identifying key contributing features to process performance, but prediction tends to be biased toward conditions in training data; they interpolate well but extrapolation is challenging. A trend has, therefore, emerged toward hybrid frameworks that combine mechanistic descriptions, offering as much descriptive power as data can support, with empirical models to address the unknowns. The result is a class of models that effectively captures training data while requiring less evaluation time compared to purely mechanistic models. It remains to be seen, however, how these frameworks will perform as digital twins. These models have also been developed with small‐scale systems in mind. Application of such methods with larger fermenters (those used in the white biotechnology space routinely reach 500 m^3^ working volume or larger) presents a challenge that is being addressed in parallel through hybrid modelling from a fluid dynamics perspective. Integrating detailed hybrid models of metabolism with hybrid descriptions of fluid dynamics offers a promising route to meaningful application at scale. This approach could yield a semi‐mechanistic representation of metabolism and the fermentation environment with improved predictive capability and relevance for digital twins, while maintaining a mathematical framework simple enough for real‐time implementation.

Computational fluid dynamics (CFD) simulations of bioreactors can provide a high‐fidelity snapshot of dissolved gas and nutrient concentration gradients in bioreactors, and thereby predict microenvironments (e.g., starvation regions, for instance, oxygen). In this special issue, De La Torre et al. (2025) engineer xylose consumption into *Yarrowia lipolytica*, providing comparative analysis of lipid production using the two primary xylose uptake pathways. Ultimately, performance is determined by oxygen availability—the xylose isomerase pathway performs better under oxygen‐limited conditions and the xylose reductase pathway under excess conditions. CFD can therefore provide a means to understand how a large‐scale system can be designed with a specific strain in mind. CFD can also be used to inform strain engineering—in this example, the xylose uptake pathway can be chosen for (or tailored to) to the microenvironments encountered in the large‐scale system.

This special issue also includes a paper that compares scales—an important application where CFD can be applied to improve understanding and increase probability of translational success. Specifically, Thiele et al. (2024) develop and scale from 1 L to 150 L a high‐cell‐density *Ralstonia eutropha* process for PHA production with tunable monomer composition. While differences were noted, performance was not dramatically different between the two scales. When scaling from 150 L to production scale, differences in the environment will be more pronounced, and metabolism‐coupled CFD simulations can be applied to understand the impact of nutrient and/or substrate‐limited microenvironments on biomass and PHA yields and productivities. The practice of proactively running simulations before scaling processes can help to de‐risk scale‐up and improve the probability of success.

## 8 ‘Begin with the Cost in Mind’: the Key to Success at Scale

Companies invest heavily in R&D to stay competitive, particularly in sectors like pharmaceuticals, bioenergy, and environmental industries. The editorial by Takors (2025) in this Special Issue highlights the urgent shift from fossil‐based industries to sustainable alternatives driven by climate change, emissions, and resource instability. Biomanufacturing is presented by Takors as a key component of a circular economy, using renewable inputs and waste streams to reduce environmental impact. A major barrier is scaling technologies from lab to industry, the known as “valley of death”, where many innovations fail to commercialize. To succeed, processes must be cost‐competitive with fossil‐based processes. For these reasons and given limited resources and high risks, effective R&D valuation models are crucial for optimizing investments and maximizing ROI. Valdivia et al. (2020) introduced the Applicable R&D Valuation (ARDV) model, tailored for large‐scale, capital‐intensive projects, such as bioenergy and biochemical production from Municipal Solid Wastes. By combining decision analysis, NPV assessment, decision tree methodologies, and Monte Carlo simulations, the ARDV model provides a practical framework for simulating progress, managing risks, and setting milestones. The model identified the key challenges in bioenergy from 2G feedstocks include improving organic matter deconstruction, high microbial fermentation efficiency, and gene discovery for novel chemical pathways.

Takors (2025) detailed that production scale is critical, particularly because large facilities benefit from economies of scales. He noted that equipment 1000‐fold larger than reference size only cost about 60 times more than the reference and that this applied for application platforms in the scale of 5–50 m^3^. Feedstock choice also matters, first‐generation inputs are common, while second‐ and third‐generation options are more sustainable but technically complex (Pfleger and Takors 2023). Then early design decisions, such as aerobic vs. anaerobic, affect efficiency and scalability. In this context it should also be noted that bioreactor design and maintaining key metrics (titers, rates, yields) are essential for scale‐up and that full exploitation of synthetic biology to engineer the most suitable strains are part of the challenge, this without forgetting that the models should include downstream processing as they may represent up to half of the cost. In this regard Valdivia et al. (2020) noticed that the judicious application of ARDV models can reduce premature policy decisions that push unready technologies to scale could undermine investor confidence and create a path to a sustainable bioeconomy marked by unmet expectations; this is particularly relevant in the fermentations technologies since United Nations and other organization aim to achieve 30% renewable industrial production by 2050. Because “Green chemistry” supports this transition by reducing fossil fuel dependence, creating rural jobs, and cutting greenhouse gas emissions, current less competitive biological production systems may need subsidies to establish the technologies and advance toward their implementation. For instance, it is known that production of polyethylene terephthalate from first‐generation sugars is twice as expensive as chemical production, but advances in transformation technologies and even use of second‐generation sugars are expected to equalize production prices while keeping environmental benefits.

Ultimately, the success of biomanufacturing relies on collaboration across biologists, bioinformaticians, engineers, economists, and business developers, among other disciplines (Ramos and Duque 2019). Progress has been made, but continued effort is essential to unlock the full potential of bioprocess innovations.

## Author Contributions

**Carolina A. Barcelos:** writing – original draft, writing – review and editing. **Juan L. Ramos:** writing – original draft, writing – review and editing, conceptualization. **Shamlan M. S. Reshamwala:** writing – original draft, writing – review and editing. **Juan Nogales:** writing – original draft, writing – review and editing. **Charles J. Foster:** writing – original draft, writing – review and editing. **Davinia Salvachúa:** conceptualization, writing – original draft, writing – review and editing. **Pavel Dvořák:** writing – original draft, writing – review and editing.

## Funding

The authors have nothing to report.

## Conflicts of Interest

The authors declare no conflicts of interest.

## Data Availability

The authors have nothing to report.
